# The Role of Serotonergic and Noradrenergic Descending Pathways on Performance-Based Cognitive Functioning at Rest and in Response to Exercise in People with Chronic Whiplash-Associated Disorders: A Randomized Controlled Crossover Study

**DOI:** 10.3390/clinpract13030063

**Published:** 2023-06-06

**Authors:** Iris Coppieters, Jo Nijs, Mira Meeus, Margot De Kooning, Emma Rheel, Eva Huysmans, Roselien Pas, Wouter Van Bogaert, Ives Hubloue, Kelly Ickmans

**Affiliations:** 1 Pain in Motion Research Group (PAIN), Department of Physiotherapy, Human Physiology and Anatomy, Faculty of Physical Education and Physiotherapy, Vrije Universiteit Brussel, 1090 Brussels, Belgium; iris.coppieters@vub.be (I.C.); margot.de.kooning@vub.be (M.D.K.); emma.rheel@vub.be (E.R.); eva.huysmans@vub.be (E.H.); roselien.pas@gmail.com (R.P.); wouter.van.bogaert@vub.be (W.V.B.); kelly.ickmans@vub.be (K.I.); 2 Department of Physical Medicine and Physiotherapy, University Hospital Brussels, 1090 Brussels, Belgium; 3 Department of Rehabilitation Sciences, Faculty of Medicine and Health Sciences, Ghent University, 9000 Ghent, Belgium; mira.meeus@uantwerpen.be; 4 Department of Health and Rehabilitation, Unit of Physiotherapy, Institute of Neuroscience and Physiology, Sahlgrenska Academy, University of Gothenburg, 405 30 Gothenburg, Sweden; 5 Vrije Universiteit Brussels, Brussels Health Campus Jette, Erasmus Building, PAIN-KIMA, Laarbeeklaan 121, BE1090 Brussels (Jette), Belgium; 6 Movant, Department of Rehabilitation Sciences and Physiotherapy, Faculty of Medicine and Health Sciences, University of Antwerp, 2610 Antwerp, Belgium; 7 Department of Experimental-Clinical and Health Psychology and Educational Sciences, Ghent University, 9000 Ghent, Belgium; 8 Department of Public Health (GEWE), Faculty of Medicine and Pharmacy, Vrije Universiteit Brussel, 1090 Brussels, Belgium; 9 Department of Emergency Medicine, University Hospital Brussels, 10090 Brussel, Belgium; ives.hubloue@uzbrussel.be; 10 Research Foundation—Flanders (FWO), 1090 Brussels, Belgium

**Keywords:** chronic whiplash-associated disorders, serotonin, norepinephrine, cognition

## Abstract

(1) Background: Dysregulation in serotonergic and noradrenergic systems may be implicated in the neurobiophysiological mechanisms underlying pain-related cognitive impairment in chronic whiplash-associated disorders (CWAD). This study aimed to unravel the role of serotonergic and noradrenergic descending pathways in cognitive functioning at rest and in response to exercise in people with CWAD. (2) Methods: 25 people with CWAD were included in this double-blind, randomized, controlled crossover study. Endogenous descending serotonergic and noradrenergic inhibitory mechanisms were modulated by using a single dose of a selective serotonin reuptake inhibitor (Citalopram) or a selective norepinephrine reuptake inhibitor (Atomoxetine). Cognitive performance was studied at rest and in response to exercise (1) without medication intake; (2) after intake of Citalopram; and (3) after intake of Atomoxetine. (3) Results: After Atomoxetine intake, selective attention improved compared with the no medication day (*p* < 0.05). In contrast, a single dose of Citalopram had no significant effect on cognitive functioning at rest. When performing pairwise comparisons, improvements in selective attention were found after exercise for the no medication condition (*p* < 0.05). In contrast, after intake of Citalopram or Atomoxetine, selective and sustained attention worsened after exercise. (4) Conclusions: A single dose of Atomoxetine improved selective attention only in one Stroop condition, and a single dose of Citalopram had no effect on cognitive functioning at rest in people with CWAD. Only without medication intake did selective attention improve in response to exercise, whereas both centrally acting medications worsened cognitive performance in response to a submaximal aerobic exercise bout in people with CWAD.

## 1. Introduction

People with chronic whiplash-associated disorders (CWAD) are characterized by persistent neck pain lasting more than three months, resulting from a whiplash injury [1]. Other associated symptoms reported by individuals with CWAD are psychological problems, difficulties with concentration and attention, and disability [2,3,4,5,6].

Cognitive problems associated with higher disability [6] and reduced quality of life [3] are perceived as very debilitating. Impairments in cognitive functioning are present in individuals with CWAD [3,6,7,8,9,10]. Nevertheless, the exact underlying mechanisms causing these cognitive dysfunctions remain unclear. Mild traumatic brain injury [11], chronic fatigue [12], litigation [12], pain intensity [7], and signs indicative of central sensitization (CS) or nociplastic pain [3,8,13] have been associated with cognitive disturbances in CWAD.

Notably, pain and cognition share common neural substrates and modulate one another reciprocally [14,15]. Pain can negatively affect cognitive performance [14], and associations have been revealed between decreased efficacy of endogenous pain inhibition and impaired cognitive functioning in people with chronic pain with features suggestive of CS [3,16]. In healthy persons, more efficient endogenous pain inhibition has been associated with better cognitive performance [3,8]. Hence, it can be hypothesized that dysfunctional endogenous pain inhibition, which is demonstrated in CWAD at rest and during exercise [17,18,19], precludes optimal cognitive capabilities in CWAD [20]. 

The neurotransmitters serotonin and norepinephrine have complex modulatory roles in pain signaling [21] and play a crucial role in endogenous pain inhibition [22]. Therefore, dysregulation of serotonin and norepinephrine systems is likely to be partly responsible for the malfunctioning of descending pain inhibitory pathways [21,23,24]. In addition, serotonin and norepinephrine systems exert profound influences on various cognitive functions such as attention, vigilance, and memory [15,25,26,27,28,29]. Accordingly, dysregulation in these monoamines may be implicated in the mechanisms underlying pain-related cognitive impairment, but research exploring this hypothesis is lacking. 

Furthermore, brain-orchestrated activation of serotonergic and noradrenergic systems also plays a key role in effective exercise-induced hypoalgesia [30]. In healthy persons, a single bout of exercise results in hypoalgesia, while it may increase pain in patients with signs of CS [18,31,32]. In addition, the exercise-induced increase in serotonin and norepinephrine [33] appears to mediate the improvement of cognitive functioning after exercise [34]. A single bout of aerobic exercise has positive effects on cognitive performance in healthy individuals [9,35,36] and on attention in patients with CWAD, as our research group previously found [9].

To unravel the biological nature of pain-related cognitive impairment, the first aim was to examine the isolated effect of activating serotonergic or noradrenergic descending pathways on performance-based cognitive functioning in people with CWAD by using a single dose of a selective serotonin reuptake inhibitor (SSRI) and a selective norepinephrine reuptake inhibitor (NRI), respectively. The second aim was to investigate the effect of activating either serotonergic or noradrenergic descending pathways on post-exercise cognitive functioning in people with CWAD. We hypothesized that activation of serotonergic and/or noradrenergic descending pathways would improve cognitive functioning both at rest and in response to exercise in people with CWAD. 

## 2. Materials and Methods

### 2.1. Study Design and Setting

**Figure 1:**
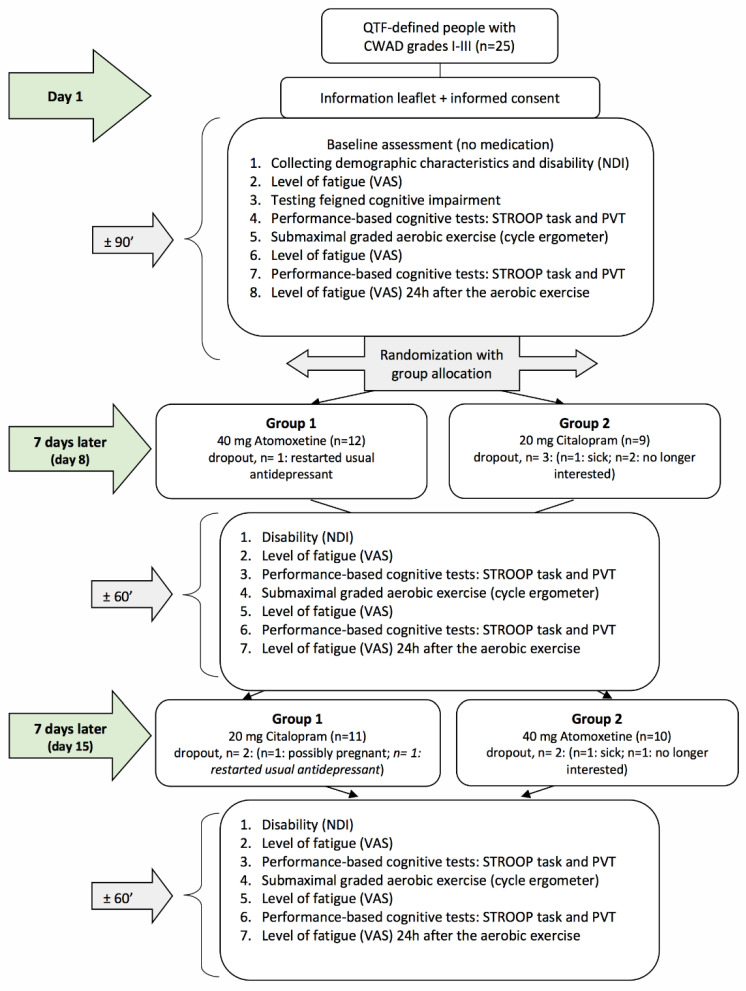
Flowchart of the randomized crossover study. QTF = Quebec Task Force; CWAD = chronic whiplash-associated disorders; VAS = visual analogue scale; PVT = psychomotor vigilance task; NDI = Neck Disability Index.

This study comprised a double-blind, randomized, controlled crossover study comparing three conditions; (1) no medication intake (baseline condition), (2) after intake of 20 mg Citalopram (SSRI), and (3) after intake of 40 mg Atomoxetine (selective NRI) (Figure 1). This study took place at the Department of Physical Medicine and Physiotherapy of the University Hospital Brussels (Belgium). The research protocol was approved by the Ethics Committee of the University Hospital Brussels/Vrije Universiteit Brussel and was in compliance with the Declaration of Helsinki. The study drugs were produced according to the Good Manufacturing Practice. All participants were thoroughly informed about the study procedures and signed a consent form prior to study enrolment. This study was registered with ClinicalTrials.gov (Identifier No. NCT01601912) and is reported in accordance with the CONSORT statement extension to randomized crossover trials [37] (Supplementary Materials).

The a-priori sample size calculation was performed with G*Power 3.1.5 and was based on the results of a study by Cook et al. [38] on the effect of a single submaximal exercise bout on cognitive performance in people with chronic fatigue syndrome, with and without comorbid fibromyalgia (a condition partly overlapping with CWAD), which found a small Partial η2 value of 0.04. It revealed that a sample size of 25 participants would provide 81% power with α = 0.05 to detect a statistically significant difference in cognitive performance pre-exercise versus postexercise.

### 2.2. Participants

People with CWAD were recruited via the Department of Physical Medicine and Physiotherapy and the Department of Emergency Medicine of the University Hospital Brussels through social media, advertisements, and a patient-support group.

Participants were eligible if they had persistent neck pain lasting at least three months resulting from a motor vehicle crash or traumatic event classifiable as WAD I, II, or III according to the Quebec Task Force criteria [39]. All participants were between 18 and 65 years old. Exclusion criteria included: (1) initial fulfillment of the WAD grade IV Quebec Task Force criteria [39]; (2) being pregnant or up to one year postnatal; (3) not being a native Dutch speaker; (4) intellectual disabilities; (5) other comorbidities that could explain the pain; (6) loss of consciousness due to the whiplash injury; (7) presence of psychiatric, metabolic, orthopedic, cardiovascular, or inflammatory disorders, and (8) presence of feigned cognitive impairment (i.e., malingering). To screen for the latter, potential participants had to complete the Rey 15-Item Memory Test for malingering during their initial study visit [40]. The description of this test can be found in a previous study of our research group [9].

Included patients were instructed to stop the use of opioid analgesics, antidepressants, and anti-epileptic medications two weeks prior to study participation. On each assessment day, the participants were asked to refrain from taking non-opioid analgesics and beta-adrenergic blocking agents; not to consume caffeine, alcohol, and nicotine; and not to undertake physical exertion. Patients were able to take non-opioid pain medication during the three-week study period but not on the assessment days.

### 2.3. Procedure

Baseline assessments were performed on the first test day without medication intake. Subsequently, cognitive performance was examined by two performance-based cognitive tests (Stroop task followed by Psychomotor Vigilance Task (PVT)). These tests were administered before and after a single submaximal aerobic exercise. Cognitive tests were performed 11 to 20 min postexercise because this yields the largest positive effects of a single exercise on cognitive performance [35]. 

After baseline assessment, an independent researcher who was not involved in the study randomly allocated the participants to one of the two groups (group 1 = day 8: selective NRI + day 15: SSRI and group 2 = day 8: SSRI + day 15: selective NRI (see Figure 1)) according to a computer-generated randomization list (http://www.randomization.com, accessed on 22 February 2023). Randomization was concealed using opaque, closed envelopes. On day 1, participants received their first single dose of medication (blinded) for day 8 and instructions regarding its administration (see *Medication administration*). One week after the baseline assessments, all participants were invited for the second time (day 8), and the same assessments were performed as on day 1. Afterward, they received their second single dose of medication (blinded) for day 15, as well as instructions regarding its administration (see *Medication administration*). One week after the second assessment, participants visited the university for the third time (day 15), and the same assessments as on day 8 were performed. Hence, the washout period was 7 days. After the final assessment, the success of participant and assessor blinding was examined by asking both to indicate group allocation, including the percentage of certainty. Reasons for study withdrawal were monitored. 

#### 2.3.1. Medication Administration

In order to ensure peak concentrations at the time of testing, all participants were instructed to take Citalopram (20 mg per os; Citalopram Sandoz^®^) four hours and Atomoxetine (40 mg per os; Strattera^®^) one and a half hours, respectively, before the scheduled start of their next appointment [41,42]. Participants were instructed not to ingest food 30 min before and after medication intake to ensure that the attainment of peak concentration was not delayed. Both pills were transparent hard-shelled capsules filled with dry white powder. The medication doses were based on the usual daily dose of Citalopram (20 mg per os) and the daily starting dose of Atomoxetine (40 mg per os) in adults.

Previous research showed that a single dose of Citalopram or Atomoxetine could alter cognitive functioning in healthy individuals and patients [43,44,45,46].

The washout period was seven days. The interval of seven days between assessment days minimizes pharmacological carry-over effects in view of the short half-life of each medication [47,48]. A single oral dose of Citalopram and Atomoxetine has, respectively, a plasma half-life time of approximately 33 h [49] and five hours [41,50].

#### 2.3.2. Submaximal Aerobic Exercise

The acute submaximal graded aerobic exercise was performed on a cycle ergometer (Kardiomed, Alzenau, Germany), with the seat adjusted appropriately for each participant. After two minutes in the resting position, the participant’s resting heart rate was measured (heart rate monitor Polar Electro Oy, Finland). The workload started at 25 W and was increased by 25 W every minute until the participant had reached the submaximal level (i.e., target heart rate, defined as 75% of the age-predicted maximal heart rate: (220 − age) × 0.75). Participants were instructed to cycle at a constant pedaling rate of approximately 60 revolutions per minute (rpm). Heart rate was recorded at the end of every minute. The exercise was terminated when participants reached their individual target heart rates. Cooling down comprised of one minute of cycling at a workload of 25 W and a rate of 60 rpm. This aerobic power index test [51] is reliable as a submaximal exercise in people with chronic pain [52].

### 2.4. Demographic Characteristics and Self-Reported Measures

Demographic characteristics and medication use were questioned. Additionally, the time of cognitive testing was noted. 

The Neck Disability Index (NDI) was used to investigate neck pain-related disability levels (0–100) [53,54]. The NDI is valid and reliable for people with chronic neck pain [55,56]. 

Participants had to indicate their present levels of fatigue by drawing a vertical line on a 100-mm visual analogue scale (VAS). This VAS was filled out for each test day: before the first performance of the cognitive tests, immediately after the exercise, and 24 h after the exercise to investigate post-exertional fatigue changes. Drawing the line at 0 mm indicates no fatigue, and drawing the line at 100 mm represents unbearable fatigue [57,58].

### 2.5. Performance-Based Cognitive Function

To investigate cognitive function, participants performed the Stroop task and the PVT. Each test began with the presentation of written instructions. Completion of this cognitive test battery took between 20 and 30 min. The Stroop task and PVT have been described in detail in our previous studies [3,9,59].

The Stroop task [60] was used to examine selective attention, choice reaction time, and cognitive inhibition. Four different intermixed conditions were used, namely, incongruent (word and ink color are different), congruent (word and ink color are the same), category (animal names written in one color), and negative priming inverse (e.g., the word “red” displayed in green font immediately followed by the word “green” displayed in red font). Negative priming is the condition where the to-be-ignored response in the first presentation becomes the subsequent relevant dimension [61].

In order to determine cognitive inhibition ability, the Stroop reaction time of the congruent condition is subtracted from the Stroop reaction time of the incongruent condition [62]. This way, an interference score is calculated, which can be interpreted as the cognitive inhibition subcomponent of executive functioning. The Stroop interference score, mean response reaction time for correct responses, and accuracy for each condition were used in further analyses.

The PVT [63] was administered to assess vigilance or sustained attention and simple reaction time. Participants were instructed to respond to a visual stimulus (red spot on a black screen) that appeared in the middle of the screen at random interstimulus intervals varying from 2 to 10 s. Participants were instructed to press the mouse button with the thumb as quickly as possible whenever they perceived the appearance of the red spot. The trial was stored as a lapse if the participant did not respond within 500 milliseconds. The mean PVT reaction time of correct responses and the number of PVT lapses were registered and used for further analyses. The PVT has good test-retest reliability [64].

### 2.6. Statistical Analysis

Statistical analyses were performed using IBM^®^SPSS^®^ Statistics 26.0. First, the normality of demographic variables was checked, and the residuals of the linear mixed models were checked for normal distribution. To investigate the comparability of pain-related disability and exercise characteristics between all assessment days, a random-intercept linear mixed models analysis was performed using a variance components covariance matrix and the factor “medication condition” with three levels (i.e., no medication, Citalopram, and Atomoxetine) as a fixed effect together with a random intercept for each patient. To investigate the comparability of levels of fatigue between and within all assessment days, a random-intercept linear mixed models analysis was performed using a variance components covariance matrix with the factor “medication condition” with the same three levels and factor “time” with three levels (i.e., VAS fatigue pre-exercise, postexercise, 24 h postexercise) and “medication x time” as fixed effects together with a random intercept for each patient.

In addition, a similar linear mixed models analysis with cognitive performance variables as outcome measures were applied, including “medication condition”, “time”, and “medication condition x time” as fixed effects together with a random intercept for each patient. For each outcome parameter, a linear mixed models analysis was applied to reveal group (i.e., medication condition)-by-time (i.e., pre-post aerobic exercise) interaction effects. Next, pairwise comparisons of the cognitive performance variables were performed for the factor “time” (pre-post aerobic exercise) within each medication condition and for the factor “medication condition” before the aerobic exercise using a Bonferroni correction to correct for multiple comparisons. Furthermore, randomization was included as a covariate in the model to test the absence of a sequence effect. *p* < 0.05 (two-sided) was considered significant.

Effect sizes of the mean differences in cognitive performance between pre-post exercise within each medication condition and effect sizes of the mean differences in cognitive performance between the no medication condition and, respectively, the Citalopram or Atomoxetine conditions before the exercise were calculated as Cohen’s *d*.

## 3. Results

### 3.1. Participant Flow, Group Characteristics, and Self-Reported Measures

Twenty-five people with CWAD (15 women, 10 men) were included in the study. Thirteen participants were randomly allocated to the Atomoxetine–Citalopram (group 1) sequence group and 12 participants were randomly allocated to the Citalopram–Atomoxetine (group 2) sequence group. No sequence, period, or first-order carryover effects are present in this crossover study. Demographic data and medication use are presented in Table 1. Only one participant took an anti-depressant (Redomex^®^), but medication intake was discontinued two weeks prior to study participation. Pain-related disability and fatigue levels are presented in Table 2. Comparable moderate pain-related disability levels were reported on all assessment days (*p* > 0.05). Furthermore, levels of fatigue were not significantly different pre-, immediately post-, and 24 h postexercise within each of the three assessment days nor between all assessment days (*p* > 0.05).

The success of assessor blinding was 100%, whereas the success of patient blinding was 96%. The dropout rate was 16%. Six patients did not complete the entire study resulting in five dropouts during the Citalopram test day and three dropouts during the Atomoxetine test day. The reasons for withdrawal from the study are presented in Figure 1. No malingering was demonstrated among the participants. Furthermore, no harm or unintended effects of the medication intake were reported. 

### 3.2. Submaximal Aerobic Exercise

Results of submaximal aerobic exercise characteristics measured during baseline, after intake of Citalopram and Atomoxetine, are listed in Table 2. Significant differences were revealed regarding the estimated mean (95% confidence interval (CI)) resting heart rate between the no medication and the Atomoxetine condition and regarding the estimated mean (95% CI) cycle duration time and maximal workload between the Citalopram and Atomoxetine assessment days (*p* < 0.05).

### 3.3. The Isolated Effect of a Single Dose of a SSRI or a Selective NRI on Cognitive Performance at Rest in People with CWAD

**Figure 2:**
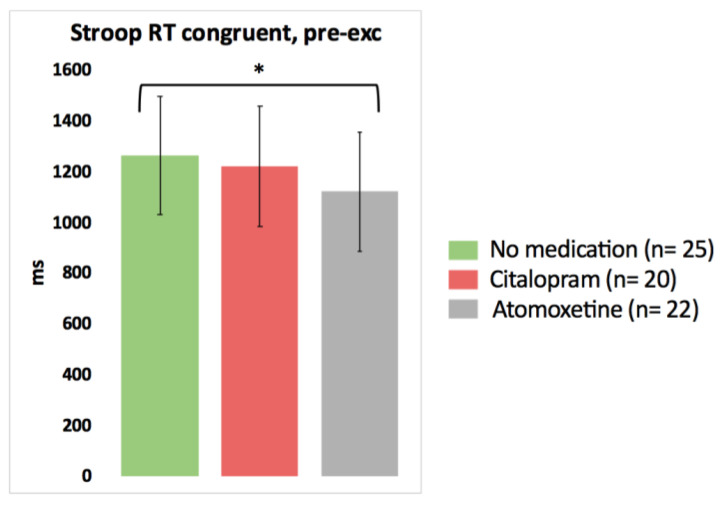
The isolated effect of activated serotonergic or noradrenergic descending pathways on cognitive performance at rest in people with chronic whiplash-associated disorders. Estimated means and 95% confidence intervals are presented. * = *p* < 0.05; exc: exercise; RT: reaction time; only significant results are presented.

Based on the pairwise comparisons of the linear mixed models analysis, after the intake of Atomoxetine, choice reaction time significantly improved for one Stroop condition (faster Stroop reaction time congruent) compared to the no medication test day (*p* = 0.048, Cohen’s *d* = −0.37) (Figure 2; detailed statistics presented in Table 3). No other significant differences in the results of the cognitive tests pre-exercise were found between the no medication condition on the one hand and the Citalopram or Atomoxetine condition on the other hand (*p* > 0.05).

### 3.4. The Effect of a Single Dose of a SSRI or a Selective NRI on Cognitive Performance in Response to Submaximal Aerobic Exercise in People with CWAD

**Figure 3:**
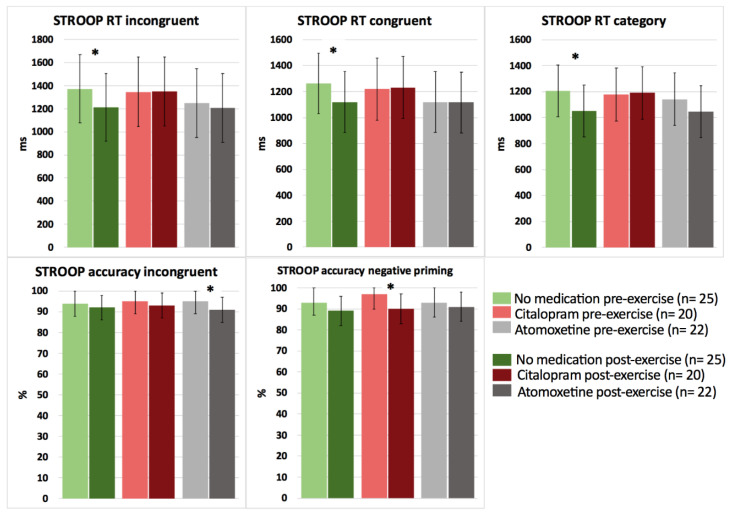
The effect of a single dose of Citalopram or Atomoxetine on cognitive performance (i.e., selective attention and choice reaction time) in response to exercise in people with chronic whiplash-associated disorders. Estimated means and 95% confidence intervals are presented. * = *p* < 0.05; RT: reaction time; only significant results are presented.

Based on the interaction effects, no significant differences were revealed between the medication conditions (no medication, Citalopram, Atomoxetine) on cognitive performance in response to a bout of acute submaximal aerobic exercise in people with CWAD (*p* > 0.05). However, when performing pairwise comparisons (pre-post submaximal exercise) within each medication condition, significant improvements in selective attention for Stroop reaction time incongruent (*p* = 0.025, *d* = −0.70) and choice reaction time for Stroop reaction time congruent (*p* = 0.018, *d* = −0.92) and category (*p* = 0.012, *d* = −0.65) were found after exercise for the no medication condition (Figure 3; detailed statistics presented in Table 4).

**Figure 4:**
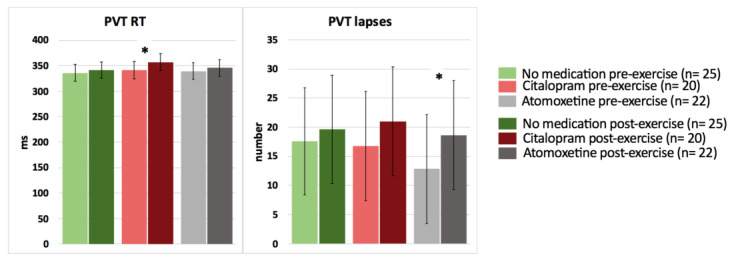
The effect of a single dose of Citalopram or Atomoxetine on cognitive performance—sustained attention and simple reaction time—in response to exercise in people with chronic whiplash-associated disorders. Estimated means and 95% confidence intervals are presented. * = *p* < 0.05; RT: reaction time; PVT: psychomotor vigilance task; only significant results are presented.

In contrast, after intake of Citalopram or Atomoxetine, both selective and sustained attention significantly worsened, and simple (PVT) reaction time significantly increased postexercise (*p* < 0.05) (Figure 3 and Figure 4; Table 4). More specifically, people with CWAD showed significantly decreased accuracy in Stroop reaction time incongruent after the submaximal exercise bout on the Atomoxetine assessment day (*p* = 0.030, *d* = −0.50). Additionally, participants showed a significantly higher number of lapses during the PVT after exercise compared to the pre-exercise result on the Atomoxetine assessment day (*p* = 0.034, *d* = 0.64) (Figure 4). After intake of Citalopram, Stroop accuracy negative priming significantly worsened after the aerobic exercise (*p* = 0.015, *d* = −0.65) (Figure 3). Furthermore, after Citalopram intake, simple reaction time significantly increased postexercise, thus sustained attention worsened (*p* = 0.021, *d* = 0.53) (Figure 4).

## 4. Discussion

This innovative study investigated the effects of a single dose of a SSRI and a selective NRI on cognitive performance at rest and in response to exercise in people with CWAD. At rest, the intake of a single dose of Atomoxetine had a positive influence on the results of the Stroop task of only one condition by decreasing the choice reaction time during the Stroop congruent condition compared to the Stroop congruent reaction time measured without Atomoxetine intake (small effect size). The latter finding of improved selective attention is in line with our hypothesis. Nevertheless, Atomoxetine had no significant isolated effect on all other cognitive performance variables in people with CWAD. Furthermore, no significant effects of a single dose of Citalopram on cognitive functioning at rest in people with CWAD could be demonstrated. It is noteworthy that the possible reported side effects of both medications that could have an influence on cognition are drowsiness, sleeping problems, and fatigue [65]. Nonetheless, an acute dose of Citalopram or Atomoxetine did not worsen cognitive performance at rest in people with CWAD compared to the no medication condition. Additionally, levels of fatigue were similar on all assessment days.

It might be possible that activation of noradrenergic transmission pre-exercise and the subsequent increased availability of norepinephrine after Atomoxetine use enhanced selective attention, but further work in this area is necessary. Citalopram intake had no significant isolated effect on cognitive performance, which is in accordance with findings in healthy persons [46,66,67].

This study provides the novel insight that the positive effects of a bout of acute aerobic exercise on selective attention and choice reaction time (medium to large effect sizes) could only be detected when no SSRI or selective NRI was taken by people with CWAD. In addition, WAD symptoms, such as pain (based on our previous study [9]) and fatigue, were not exacerbated either immediately or 24 h postexercise. 

A positive influence of acute aerobic exercise on cognitive functioning was, on the one hand, hypothesized because this has been demonstrated in patients with chronic fatigue syndrome [68] and healthy people [35,36]. Furthermore, evidence is available in various chronic pain conditions that exercise therapy has a positive effect on cognitive functioning [69,70,71,72,73]. On the other hand, some evidence exists for the worsening of symptoms following physical exertion in women with CWAD [18]. Nevertheless, in a recent study, patients with CWAD did not perceive increased pain sensitivity following aerobic exercise [74].

The mechanisms that could explain the observed beneficial effects of acute aerobic exercise on cognitive functioning are presumed to be driven by physiological responses to exercise. These responses comprise changes in heart rate and plasma catecholamines, increased levels of growth factors such as brain-derived neurotrophic factor (BDNF) [35], and brain neurotransmitters such as norepinephrine, serotonin, and dopamine, mediating the exercise-induced enhancement of cognition [33,34].

The intake of a single dose of Atomoxetine resulted in a worsening of Stroop accuracy reaction time incongruent and in more errors of omission during the PVT in response to the acute exercise bout (medium effect size). Similarly, a single dose of Citalopram resulted in a worsening of Stroop accuracy reaction time (negative priming) and gave rise to diminished sustained attention after the exercise compared to the pre-exercise condition (medium effect size). The latter results are not according to our hypotheses. Because changes in, for example, BDNF, serotonin, and norepinephrine levels in response to the exercise performance were not measured, we cannot state which mechanisms accounted for the observed changes in cognitive functioning following the exercise.

Possibly, the acute medication-induced increased levels of serotonin and norepinephrine in the brain had a negative influence on the mediating effect of these monoamines on cognitive performance in response to exercise. On the other hand, it could be that the single acute dose of both centrally acting drugs was not adequate to successfully activate serotonergic and noradrenergic descending pathways in response to exercise and hence, to obtain positive effects on postexercise cognition. Therefore, the present study has limited clinical implications. It can be concluded that clinicians are advised not to use single doses of Citalopram or Atomoxetine to improve cognitive performance at rest or in response to exercise in people with CWAD.

### Limitations and Recommendations for Further Research

The following limitations should be taken into account. As we could not demonstrate significant interaction effects, and a small sample size increases the risk of committing a type II error, further research with a larger sample size is warranted before firm conclusions can be drawn.

This study only investigated the effects of a single dose of Citalopram and Atomoxetine in people with CWAD. In order to ensure peak concentrations at the time of testing, all participants were instructed to take Citalopram (20 mg per os; Citalopram Sandoz^®^) four hours and Atomoxetine (40 mg per os; Strattera^®^) one and a half hours, respectively, before the scheduled start of their appointment [41,42]. However, people with chronic pain usually take these medications over a long period of time. Perhaps, these medications should be taken for a longer period of time before exerting positive effects on the influence that exercise exerts on cognitive functioning. Indeed, the onset of action of Citalopram for depression is approximately 1 to 4 weeks, and the complete response may take 8–12 weeks after initiation. Future work should examine whether such long-term administration of Citalopram has different effects, as observed here. Furthermore, this trial is not placebo-controlled. We did not include a condition with a placebo medication which could have been useful to blind participants also for the test day without medication and to enhance insights into underlying mechanisms. Originally, we intended to include a placebo group, but the ethical committee did not allow us to do that.

The improved selective attention combined with the absence of post-exertional aggravation of symptoms in response to the acute aerobic exercise in individuals with CWAD indicates the relevance of further randomized controlled trials to study the effects of graded aerobic exercise therapy on cognitive functioning.

## 5. Conclusions

In conclusion, a single dose of Atomoxetine improved selective attention only in one Stroop condition, and a single dose of Citalopram had no effect on cognitive functioning at rest in people with CWAD. Only without medication intake did selective attention improve in response to exercise, whereas both centrally acting medications worsened cognitive performance in response to a submaximal aerobic exercise bout in people with CWAD. Further research with larger sample sizes is warranted. Examining the influence of the long-term use of selective serotonin reuptake inhibitors and selective norepinephrine reuptake inhibitors on the physiological response to exercise training and subsequent effects on cognitive functioning in people with chronic pain is a future research avenue.

## Figures and Tables

**Table 1:** Demographic data in people with CWAD (*n* = 25) separately for each sequence.

	Citalopram—Atomoxetine (*n* = 12)	Atomoxetine—Citalopram (*n* = 13)
Age, y	42.7 (11.6)	38.9 (9.9)
Women, *n*	7 (58.3)	8 (62)
Body mass, kg	71.8 (9.1)	75.7 (18.2)
Height, cm	170.8 (9.1)	172.5 (8.7)
BMI, kg/m^2^	24.6 (2.2)	25.2 (4.2)
Disease duration, months	89.4 (109.7)	23.4 (29.7)
Occupational situation, *n*	4 inactive (33.3); 3 part-time (25); 5 full-time (41.7); 0 students (0); 0 retired (0)	5 inactive (38.5); 3 part-time (23.1); 3 full-time (23.1); 2 students (15.4); 0 retired (0)
Education level, *n*	0 primary education (0); 4 secondary education (33.3) 7 bachelor’s degree (58.3); 1 master’s degree (8.3); 0 PhD (0)	0 primary education (0); 5 secondary education (38.5) 4 bachelor’s degree (30.8); 2 master’s degree (15.4); 0 PhD (0)
Time of cognitive testing, *n*	0 early morning (0); 5 late morning (41.7); 3 early afternoon (25) 0 late afternoon (0); 4 evening (33.3)	0 early morning (0); 3 late morning (23.1); 5 early afternoon (38.5) 2 late afternoon (15.4); 3 evening (23.1)
Antidepressants, *n*	0 (0)	1 (7.7) ^a^
Analgesics, *n*	5 (41.7) ^a^	3 (23.1) ^a^
Anti-epileptics, *n*	0 (0)	0 (0)
Legal conflict, *n*	6 no (50); 0 employer (0); 4 insurance company (33.3) 1 employer and insurance company (8.3)	7 no (53.9); 1 employer (7.7); 5 insurance company (38.5) 0 employer and insurance company (0)
Malingering/feigned cognitive impairment, *n*	0 (0)	0 (0)
Successful blinding of patients, %	96	
Successful blinding of assessors, %	100	

Values are presented as mean (SD) for data which were normally distributed or number (%) for categorical data. ^a^ All participants discontinued medication intake 2 weeks prior to study participation and on the test day (except for Citalopram or Atomoxetine according to randomization). CWAD = chronic whiplash-associated disorders, VAS = visual analogue scale, y = years.

**Table 2:** Self-reported pain-related disability and fatigue levels, and exercise characteristics in people with CWAD (*n* = 25).

	No Medication	After Intake Citalopram	After Intake Atomoxetine	*p* Value ^†^
Neck Disability Index, /100	40.2 (35.0–45.5)	38.7 (33.3–44.1)	38.7 (33.4–44.1)	>0.05
VAS fatigue, mm (/100): pre-exercise	45.5 (32.5–58.5)	48.3 (34.8–61.8)	51.6 (38.3–64.9)	>0.05 ^‡^
VAS fatigue, mm (/100): post-exercise	46.8 (33.8–59.8)	43.4 (30.0–56.9)	45.2 (31.9–58.5)	>0.05 ^‡^
VAS fatigue, mm (/100): 24 h post-exercise	53.0 (39.9–66.1)	50.7 (37.3–64.2)	45.2 (31.9–58.5)	>0.05 ^‡^
Submaximal aerobic exercise characteristics				
Resting heart rate, beats per minute	83.0 ^§^ (77.8–88.1)	84.7 (79.1–90.4)	91.6 ^§^ (86.1–97.1)	<0.05 ^§^
Duration aerobic cycling exercise, minutes	4.8 (4.3–5.3)	5.1 ^§^ (4.6–5.7)	4.5 ^§^ (3.9–5.1)	<0.05 ^§^
Max Wattage	120 (106.5–133.5)	128.1 ^§^ (113.9–142.2)	112.3 ^§^ (98.4–126.2)	<0.05 ^§^

^†^ Statistical analyses were performed using linear mixed models analyses; estimated means and 95% confidence intervals are presented. ^‡^ Within and between each medication condition there were no significant differences between levels of fatigue. ^§^ There were significant differences between both indicated conditions (differences between all conditions were examined). CWAD = chronic whiplash-associated disorders, VAS = visual analogue scale, No medication = baseline condition without medication intake. N = 25 for the no medication condition, *n* = 20 for the Citalopram condition, *n* = 22 for the Atomoxetine condition.

**Table 3:** Cognitive performance in people with CWAD in three conditions: baseline without medication intake (*n* = 25), after intake of Citalopram (SSRI), and after intake of Atomoxetine (selective NRI).

Cognitive Performance Variable	Time Condition	Medication Condition	Estimated Means	95% Confidence Interval		Medication Conditions (No Medication Versus Citalopram or Atomoxetine)		Estimated Mean Difference	*p* Value	Cohen’s d Effect Size
				Lower bound	Upper bound	Pre-exc				
	Pre-exc	No medication	1373.09	1078.38	1667.80	No medication	Citalopram	26.03	1	−0.04
		Citalopram	1347.06	1047.51	1646.61	No medication	Atomoxetine	123.05	0.200	−0.26
		Atomoxetine	1250.04	952.56	1547.53					
	Pre-exc	No medication	1262.15	1028.75	1495.56	No medication	Citalopram	43.148	1	−0.09
		Citalopram	1219.01	981.19	1456.82					
		Atomoxetine	1118.93	883.00	1354.87	No medication	Atomoxetine	143.22	0.048	−0.37
	Pre-exc	No medication	1206.95	1008.70	1405.19	No medication	Citalopram	27.43	1	−0.06
		Citalopram	1179.52	975.85	1383.19					
		Atomoxetine	1141.57	940.22	1342.93	No medication	Atomoxetine	65.38	0.614	−0.15
	Pre-exc	No medication	110.94	24.79	197.09	No medication	Citalopram	−20.58	1	0.07
		Citalopram	131.52	40.40	222.64					
		Atomoxetine	130.41	41.42	219.40	No medication	Atomoxetine	−19.47	1	0.07
	Pre-exc	No medication	1336.26	1052.46	1620.06	No medication	Citalopram	−68.64	0.830	0.10
		Citalopram	1404.90	1114.97	1694.83					
		Atomoxetine	1219.71	932.39	1507.02	No medication	Atomoxetine	116.55	0.308	−0.31
	Pre-exc	No medication	94	88	100	No medication	Citalopram	−1	1	0.21
		Citalopram	95	89	100					
		Atomoxetine	95	89	100	No medication	Atomoxetine	−1	1	0.12
	Pre-exc	No medication	99	97	100	No medication	Citalopram	1	1	0.00
		Citalopram	99	97	100					
		Atomoxetine	99	97	100	No medication	Atomoxetine	0	1	0.00
	Pre-exc	No medication	99	97	100	No medication	Citalopram	0	1	0.00
		Citalopram	99	97	100					
		Atomoxetine	99	97	100	No medication	Atomoxetine	0	1	0.00
	Pre-exc	No medication	93	87	100	No medication	Citalopram	−4	0.318	0.37
		Citalopram	97	90	100					
		Atomoxetine	93	86	100	No medication	Atomoxetine	0	1	0.00
	Pre-exc	No medication	336.12	320.03	352.21	No medication	Citalopram	−5.28	0.848	0.15
		Citalopram	341.40	324.64	358.17					
		Atomoxetine	339.55	323.07	356.03	No medication	Atomoxetine	−3.43	1	0.12
	Pre-exc	No medication	17.62	8.41	26.83	No medication	Citalopram	0.85	1	−0.11
		Citalopram	16.77	7.36	26.18					
		Atomoxetine	12.85	3.48	22.21	No medication	Atomoxetine	4.77	0.148	−0.30

Abbreviations: CWAD: chronic whiplash-associated disorders; PVT: psychomotor vigilance task; exc: exercise; *n*: number of patients; SSRI = selective serotonin reuptake inhibitor; NRI: norepinephrine reuptake inhibitor; no medication: baseline condition without medication intake. Statistical analyses were performed using linear mixed models analyses. Pairwise comparisons using Bonferroni correction to correct for multiple comparisons. Significant differences are presented in Bold. Cohen’s *d* is interpreted as “very large” (>1.3), “large” (0.80–1.29), “medium” (0.50–0.79), “small” (0.20–0.49), and “negligible” (<0.20). *n* = 25 for the no medication condition, *n* = 20 for the Citalopram condition, *n* = 22 for the Atomoxetine condition.

**Table 4:** Cognitive performance in people with CWAD before and after a single submaximal aerobic exercise bout in three conditions: baseline without medication intake (*n* = 25), after intake of Citalopram (SSRI), and after intake of Atomoxetine (selective NRI).

Cognitive Performance Variable	Medication Condition	Time Condition	Estimated Means	95% Confidence Interval			Estimated Mean Difference	*p* Value	Cohen’s d Effect Size
				Lower bound	Upper bound				
	No medication	Pre-exc	1373.09	1078.38	1667.80	Pre-Post exc	159.67	0.025	−0.70
		Post-exc	1213.42	918.71	1508.13				
	Citalopram	Pre-exc	1347.06	1047.51	1646.61	Pre-Post exc	−3.83	0.961	0.02
		Post-exc	1350.89	1051.34	1650.44				
	Atomoxetine	Pre-exc	1250.04	952.56	1547.53	Pre-Post exc	43.66	0.562	−0.70
		Post-exc	1206.38	908.90	1503.87				
	No medication	Pre-exc	1262.15	1028.75	1495.56	Pre-Post exc	143.58	0.018	−0.92
		Post-exc	1118.58	885.17	1351.98				
	Citalopram	Pre-exc	1219.01	981.19	1456.82	Pre-Post exc	−12.98	0.846	0.06
		Post-exc	1231.99	994.18	1469.80				
	Atomoxetine	Pre-exc	1118.93	883.00	1354.87	Pre-Post exc	3.14	0.961	−0.03
		Post-exc	1115.80	879.86	1351.73				
	No medication	Pre-exc	1206.95	1008.70	1405.19	Pre-Post exc	155.39	0.012	−0.65
		Post-exc	1051.56	853.31	1249.81				
	Citalopram	Pre-exc	1179.52	975.85	1383.19	Pre-Post exc	−10.08	0.883	0.05
		Post-exc	1189.60	985.93	1393.27				
	Atomoxetine	Pre-exc	1141.57	940.22	1342.93	Pre-Post exc	95.57	0.144	−0.65
		Post-exc	1046.00	844.64	1247.35				
	No medication	Pre-exc	110.94	24.79	197.09	Pre-Post exc	16.09	0.677	−0.11
		Post-exc	94.85	8.70 40.40	180.99 222.64				
	Citalopram	Pre-exc	131.52			Pre-Post exc	9.16	0.832	−0.05
		Post-exc	122.36	31.25	213.48				
	Atomoxetine	Pre-exc	130.41	41.42	219.40	Pre-Post exc	40.52	0.326	−0.29
		Post-exc	89.89	0.90	178.88				
	No medication	Pre-exc	1336.26	1052.46	1620.06	Pre-Post exc	153.34	0.051	−0.71
		Post-exc	1182.92	899.12	1466.72				
	Citalopram	Pre-exc	1404.90	1114.97	1694.83	Pre-Post exc	105.28	0.228	−0.30
		Post-exc	1299.62	1009.69	1589.55				
	Atomoxetine	Pre-exc	1219.71	932.39	1507.02	Pre-Post exc	20.72	0.803	−0.21
		Post-exc	1198.99	911.67	1486.30				
	No medication	Pre-exc	94	88	100	Pre-Post exc	2	0.196	−0.22
		Post-exc	92	86	98				
	Citalopram	Pre-exc	95	89	100	Pre-Post exc	2	0.213	−0.34
		Post-exc	93	87	99				
	Atomoxetine	Pre-exc	95	89	100	Pre-Post exc	4	0.030	−0.50
		Post-exc	91	85	97				
	No medication	Pre-exc	99	97	100	Pre-Post exc	1	0.387	−0.31
		Post-exc	98	96	100				
	Citalopram	Pre-exc	99	97	100	Pre-Post exc	2	0.055	−0.32
		Post-exc	96	94	98				
	Atomoxetine	Pre-exc	99	97	100	Pre-Post exc	0	0.614	−0.39
		Post-exc	98	96	100				
	No medication	Pre-exc	99	97	100	Pre-Post exc	1	0.478	−0.19
		Post-exc	98	96	100				
	Citalopram	Pre-exc	99	97	100	Pre-Post exc	3	0.102	−0.27
		Post-exc	96	94	99				
	Atomoxetine	Pre-exc	99	97	100	Pre-Post exc	2	0.132	−0.31
		Post-exc	97	94	99				
	No medication	Pre-exc	93	87	100	Pre-Post exc	4	0.128	−0.25
		Post-exc	89	82	96				
	Citalopram	Pre-exc	97	90	100	Pre-Post exc	7.3	0.015	−0.65
		Post-exc	90	83	97				
	Atomoxetine	Pre-exc	93	86	100	Pre-Post exc	2	0.420	−0.25
		Post-exc	91	84	98				
	No medication	Pre-exc	336.12	320.03	352.21	Pre-Post exc	−5.58	0.363	0.26
		Post-exc	341.70	325.60	357.79				
	Citalopram	Pre-exc	341.40	324.64	358.17	Pre-Post exc	−15.97	0.021	0.53
		Post-exc	357.38	340.61	374.14				
	Atomoxetine	Pre-exc	339.55	323.07	356.03	Pre-Post exc	−6.16	0.346	0.32
		Post-exc	345.71	329.23	362.19				
	No medication	Pre-exc	17.62	8.41	26.83	Pre-Post exc	−2.04	0.426	0.34
		Post-exc	19.66	10.39	28.93				
	Citalopram	Pre-exc	16.77	7.36	26.18	Pre-Post exc	−4.25	0.128	0.27
		Post-exc	21.01	11.65	30.38				
	Atomoxetine	Pre-exc	12.85	3.48	22.21	Pre-Post exc	−5.81	0.034	0.64
		Post-exc	18.65	9.33	27.98				

Abbreviations: CWAD: chronic whiplash-associated disorders; PVT: psychomotor vigilance task; exc: exercise; *n*: number of patients; SSRI = selective serotonin reuptake inhibitor; NRI: norepinephrine reuptake inhibitor. Interference score = cognitive inhibition (Stroop reaction time incongruent minus Stroop reaction time congruent); negative priming = the condition where the to-be-ignored response in the first presentation becomes the subsequent relevant dimension; no medication: baseline condition without medication intake. Statistical analyses were performed using linear mixed models analyses. Pairwise comparisons using Bonferroni correction to correct for multiple comparisons. Significant differences are presented in Bold. Cohen’s *d* is interpreted as “very large” (>1.3), “large” (0.80–1.29), “medium” (0.50–0.79), “small” (0.20–0.49), “negligible” (<0.20). *n* = 25 for the no medication condition, *n* = 20 for the Citalopram condition, *n* = 22 for the Atomoxetine condition.

## Data Availability

Please contact the corresponding author to obtain the research data. All requests for obtaining the research data will be considered by the research team.

## Supplementary Materials

The following are available online at https://www.mdpi.com/article/10.3390/clinpract13030063/s1, CONSORT 2010 checklist of information to include when reporting a randomized trial.
